# Inclusive quarkonium production at forward rapidity in pp collisions at $\mathbf {\sqrt{s}=8}~$TeV

**DOI:** 10.1140/epjc/s10052-016-3987-y

**Published:** 2016-04-05

**Authors:** J. Adam, D. Adamová, M. M. Aggarwal, G. Aglieri Rinella, M. Agnello, N. Agrawal, Z. Ahammed, S. U. Ahn, S. Aiola, A. Akindinov, S. N. Alam, D. Aleksandrov, B. Alessandro, D. Alexandre, R. Alfaro Molina, A. Alici, A. Alkin, J. R. M. Almaraz, J. Alme, T. Alt, S. Altinpinar, I. Altsybeev, C. Alves Garcia Prado, C. Andrei, A. Andronic, V. Anguelov, J. Anielski, T. Antičić, F. Antinori, P. Antonioli, L. Aphecetche, H. Appelshäuser, S. Arcelli, R. Arnaldi, O. W. Arnold, I. C. Arsene, M. Arslandok, B. Audurier, A. Augustinus, R. Averbeck, M. D. Azmi, A. Badalà, Y. W. Baek, S. Bagnasco, R. Bailhache, R. Bala, A. Baldisseri, R. C. Baral, A. M. Barbano, R. Barbera, F. Barile, G. G. Barnaföldi, L. S. Barnby, V. Barret, P. Bartalini, K. Barth, J. Bartke, E. Bartsch, M. Basile, N. Bastid, S. Basu, B. Bathen, G. Batigne, A. Batista Camejo, B. Batyunya, P. C. Batzing, I. G. Bearden, H. Beck, C. Bedda, N. K. Behera, I. Belikov, F. Bellini, H. Bello Martinez, R. Bellwied, R. Belmont, E. Belmont-Moreno, V. Belyaev, G. Bencedi, S. Beole, I. Berceanu, A. Bercuci, Y. Berdnikov, D. Berenyi, R. A. Bertens, D. Berzano, L. Betev, A. Bhasin, I. R. Bhat, A. K. Bhati, B. Bhattacharjee, J. Bhom, L. Bianchi, N. Bianchi, C. Bianchin, J. Bielčík, J. Bielčíková, A. Bilandzic, R. Biswas, S. Biswas, S. Bjelogrlic, J. T. Blair, D. Blau, C. Blume, F. Bock, A. Bogdanov, H. Bøggild, L. Boldizsár, M. Bombara, J. Book, H. Borel, A. Borissov, M. Borri, F. Bossú, E. Botta, S. Böttger, C. Bourjau, P. Braun-Munzinger, M. Bregant, T. Breitner, T. A. Broker, T. A. Browning, M. Broz, E. J. Brucken, E. Bruna, G. E. Bruno, D. Budnikov, H. Buesching, S. Bufalino, P. Buncic, O. Busch, Z. Buthelezi, J. B. Butt, J. T. Buxton, D. Caffarri, X. Cai, H. Caines, L. Calero Diaz, A. Caliva, E. Calvo Villar, P. Camerini, F. Carena, W. Carena, F. Carnesecchi, J. Castillo Castellanos, A. J. Castro, E. A. R. Casula, C. Ceballos Sanchez, J. Cepila, P. Cerello, J. Cerkala, B. Chang, S. Chapeland, M. Chartier, J. L. Charvet, S. Chattopadhyay, S. Chattopadhyay, V. Chelnokov, M. Cherney, C. Cheshkov, B. Cheynis, V. Chibante Barroso, D. D. Chinellato, S. Cho, P. Chochula, K. Choi, M. Chojnacki, S. Choudhury, P. Christakoglou, C. H. Christensen, P. Christiansen, T. Chujo, S. U. Chung, C. Cicalo, L. Cifarelli, F. Cindolo, J. Cleymans, F. Colamaria, D. Colella, A. Collu, M. Colocci, G. Conesa Balbastre, Z. Conesa del Valle, M. E. Connors, J. G. Contreras, T. M. Cormier, Y. Corrales Morales, I. Cortés Maldonado, P. Cortese, M. R. Cosentino, F. Costa, P. Crochet, R. Cruz Albino, E. Cuautle, L. Cunqueiro, T. Dahms, A. Dainese, A. Danu, D. Das, I. Das, S. Das, A. Dash, S. Dash, S. De, A. De Caro, G. de Cataldo, C. de Conti, J. de Cuveland, A. De Falco, D. De Gruttola, N. De Marco, S. De Pasquale, A. Deisting, A. Deloff, E. Dénes, C. Deplano, P. Dhankher, D. Di Bari, A. Di Mauro, P. Di Nezza, M. A. Diaz Corchero, T. Dietel, P. Dillenseger, R. Divià, Ø. Djuvsland, A. Dobrin, D. Domenicis Gimenez, B. Dönigus, O. Dordic, T. Drozhzhova, A. K. Dubey, A. Dubla, L. Ducroux, P. Dupieux, R. J. Ehlers, D. Elia, H. Engel, E. Epple, B. Erazmus, I. Erdemir, F. Erhardt, B. Espagnon, M. Estienne, S. Esumi, J. Eum, D. Evans, S. Evdokimov, G. Eyyubova, L. Fabbietti, D. Fabris, J. Faivre, A. Fantoni, M. Fasel, L. Feldkamp, A. Feliciello, G. Feofilov, J. Ferencei, A. Fernández Téllez, E. G. Ferreiro, A. Ferretti, A. Festanti, V. J. G. Feuillard, J. Figiel, M. A. S. Figueredo, S. Filchagin, D. Finogeev, F. M. Fionda, E. M. Fiore, M. G. Fleck, M. Floris, S. Foertsch, P. Foka, S. Fokin, E. Fragiacomo, A. Francescon, U. Frankenfeld, U. Fuchs, C. Furget, A. Furs, M. Fusco Girard, J. J. Gaardhøje, M. Gagliardi, A. M. Gago, M. Gallio, D. R. Gangadharan, P. Ganoti, C. Gao, C. Garabatos, E. Garcia-Solis, C. Gargiulo, P. Gasik, E. F. Gauger, M. Germain, A. Gheata, M. Gheata, P. Ghosh, S. K. Ghosh, P. Gianotti, P. Giubellino, P. Giubilato, E. Gladysz-Dziadus, P. Glässel, D. M. Goméz Coral, A. Gomez Ramirez, V. Gonzalez, P. González-Zamora, S. Gorbunov, L. Görlich, S. Gotovac, V. Grabski, O. A. Grachov, L. K. Graczykowski, K. L. Graham, A. Grelli, A. Grigoras, C. Grigoras, V. Grigoriev, A. Grigoryan, S. Grigoryan, B. Grinyov, N. Grion, J. M. Gronefeld, J. F. Grosse-Oetringhaus, J.-Y. Grossiord, R. Grosso, F. Guber, R. Guernane, B. Guerzoni, K. Gulbrandsen, T. Gunji, A. Gupta, R. Gupta, R. Haake, Ø. Haaland, C. Hadjidakis, M. Haiduc, H. Hamagaki, G. Hamar, J. W. Harris, A. Harton, D. Hatzifotiadou, S. Hayashi, S. T. Heckel, M. Heide, H. Helstrup, A. Herghelegiu, G. Herrera Corral, B. A. Hess, K. F. Hetland, H. Hillemanns, B. Hippolyte, R. Hosokawa, P. Hristov, M. Huang, T. J. Humanic, N. Hussain, T. Hussain, D. Hutter, D. S. Hwang, R. Ilkaev, M. Inaba, M. Ippolitov, M. Irfan, M. Ivanov, V. Ivanov, V. Izucheev, P. M. Jacobs, M. B. Jadhav, S. Jadlovska, J. Jadlovsky, C. Jahnke, M. J. Jakubowska, H. J. Jang, M. A. Janik, P. H. S. Y. Jayarathna, C. Jena, S. Jena, R. T. Jimenez Bustamante, P. G. Jones, H. Jung, A. Jusko, P. Kalinak, A. Kalweit, J. Kamin, J. H. Kang, V. Kaplin, S. Kar, A. Karasu Uysal, O. Karavichev, T. Karavicheva, L. Karayan, E. Karpechev, U. Kebschull, R. Keidel, D. L. D. Keijdener, M. Keil, M. Mohisin Khan, P. Khan, S. A. Khan, A. Khanzadeev, Y. Kharlov, B. Kileng, D. W. Kim, D. J. Kim, D. Kim, H. Kim, J. S. Kim, M. Kim, M. Kim, S. Kim, T. Kim, S. Kirsch, I. Kisel, S. Kiselev, A. Kisiel, G. Kiss, J. L. Klay, C. Klein, J. Klein, C. Klein-Bösing, S. Klewin, A. Kluge, M. L. Knichel, A. G. Knospe, T. Kobayashi, C. Kobdaj, M. Kofarago, T. Kollegger, A. Kolojvari, V. Kondratiev, N. Kondratyeva, E. Kondratyuk, A. Konevskikh, M. Kopcik, M. Kour, C. Kouzinopoulos, O. Kovalenko, V. Kovalenko, M. Kowalski, G. Koyithatta Meethaleveedu, I. Králik, A. Kravčáková, M. Kretz, M. Krivda, F. Krizek, E. Kryshen, M. Krzewicki, A. M. Kubera, V. Kučera, C. Kuhn, P. G. Kuijer, A. Kumar, J. Kumar, L. Kumar, S. Kumar, P. Kurashvili, A. Kurepin, A. B. Kurepin, A. Kuryakin, M. J. Kweon, Y. Kwon, S. L. La Pointe, P. La Rocca, P. Ladron de Guevara, C. Lagana Fernandes, I. Lakomov, R. Langoy, C. Lara, A. Lardeux, A. Lattuca, E. Laudi, R. Lea, L. Leardini, G. R. Lee, S. Lee, F. Lehas, R. C. Lemmon, V. Lenti, E. Leogrande, I. León Monzón, H. León Vargas, M. Leoncino, P. Lévai, S. Li, X. Li, J. Lien, R. Lietava, S. Lindal, V. Lindenstruth, C. Lippmann, M. A. Lisa, H. M. Ljunggren, D. F. Lodato, P. I. Loenne, V. Loginov, C. Loizides, X. Lopez, E. López Torres, A. Lowe, P. Luettig, M. Lunardon, G. Luparello, A. Maevskaya, M. Mager, S. Mahajan, S. M. Mahmood, A. Maire, R. D. Majka, M. Malaev, I. Maldonado Cervantes, L. Malinina, D. Mal’Kevich, P. Malzacher, A. Mamonov, V. Manko, F. Manso, V. Manzari, M. Marchisone, J. Mareš, G. V. Margagliotti, A. Margotti, J. Margutti, A. Marín, C. Markert, M. Marquard, N. A. Martin, J. Martin Blanco, P. Martinengo, M. I. Martínez, G. Martínez García, M. Martinez Pedreira, A. Mas, S. Masciocchi, M. Masera, A. Masoni, L. Massacrier, A. Mastroserio, A. Matyja, C. Mayer, J. Mazer, M. A. Mazzoni, D. Mcdonald, F. Meddi, Y. Melikyan, A. Menchaca-Rocha, E. Meninno, J. Mercado Pérez, M. Meres, Y. Miake, M. M. Mieskolainen, K. Mikhaylov, L. Milano, J. Milosevic, L. M. Minervini, A. Mischke, A. N. Mishra, D. Miśkowiec, J. Mitra, C. M. Mitu, N. Mohammadi, B. Mohanty, L. Molnar, L. Montaño Zetina, E. Montes, D. A. Moreira De Godoy, L. A. P. Moreno, S. Moretto, A. Morreale, A. Morsch, V. Muccifora, E. Mudnic, D. Mühlheim, S. Muhuri, M. Mukherjee, J. D. Mulligan, M. G. Munhoz, R. H. Munzer, S. Murray, L. Musa, J. Musinsky, B. Naik, R. Nair, B. K. Nandi, R. Nania, E. Nappi, M. U. Naru, H. Natal da Luz, C. Nattrass, K. Nayak, T. K. Nayak, S. Nazarenko, A. Nedosekin, L. Nellen, F. Ng, M. Nicassio, M. Niculescu, J. Niedziela, B. S. Nielsen, S. Nikolaev, S. Nikulin, V. Nikulin, F. Noferini, P. Nomokonov, G. Nooren, J. C. C. Noris, J. Norman, A. Nyanin, J. Nystrand, H. Oeschler, S. Oh, S. K. Oh, A. Ohlson, A. Okatan, T. Okubo, L. Olah, J. Oleniacz, A. C. Oliveira Da Silva, M. H. Oliver, J. Onderwaater, C. Oppedisano, R. Orava, A. Ortiz Velasquez, A. Oskarsson, J. Otwinowski, K. Oyama, M. Ozdemir, Y. Pachmayer, P. Pagano, G. Paić, S. K. Pal, J. Pan, A. K. Pandey, P. Papcun, V. Papikyan, G. S. Pappalardo, P. Pareek, W. J. Park, S. Parmar, A. Passfeld, V. Paticchio, R. N. Patra, B. Paul, H. Pei, T. Peitzmann, H. Pereira Da Costa, E. Pereira De Oliveira Filho, D. Peresunko, C. E. Pérez Lara, E. Perez Lezama, V. Peskov, Y. Pestov, V. Petráček, V. Petrov, M. Petrovici, C. Petta, S. Piano, M. Pikna, P. Pillot, O. Pinazza, L. Pinsky, D. B. Piyarathna, M. Płoskoń, M. Planinic, J. Pluta, S. Pochybova, P. L. M. Podesta-Lerma, M. G. Poghosyan, B. Polichtchouk, N. Poljak, W. Poonsawat, A. Pop, S. Porteboeuf-Houssais, J. Porter, J. Pospisil, S. K. Prasad, R. Preghenella, F. Prino, C. A. Pruneau, I. Pshenichnov, M. Puccio, G. Puddu, P. Pujahari, V. Punin, J. Putschke, H. Qvigstad, A. Rachevski, S. Raha, S. Rajput, J. Rak, A. Rakotozafindrabe, L. Ramello, F. Rami, R. Raniwala, S. Raniwala, S. S. Räsänen, B. T. Rascanu, D. Rathee, K. F. Read, K. Redlich, R. J. Reed, A. Rehman, P. Reichelt, F. Reidt, X. Ren, R. Renfordt, A. R. Reolon, A. Reshetin, J.-P. Revol, K. Reygers, V. Riabov, R. A. Ricci, T. Richert, M. Richter, P. Riedler, W. Riegler, F. Riggi, C. Ristea, E. Rocco, M. Rodríguez Cahuantzi, A. Rodriguez Manso, K. Røed, E. Rogochaya, D. Rohr, D. Röhrich, R. Romita, F. Ronchetti, L. Ronflette, P. Rosnet, A. Rossi, F. Roukoutakis, A. Roy, C. Roy, P. Roy, A. J. Rubio Montero, R. Rui, R. Russo, E. Ryabinkin, Y. Ryabov, A. Rybicki, S. Sadovsky, K. Šafařík, B. Sahlmuller, P. Sahoo, R. Sahoo, S. Sahoo, P. K. Sahu, J. Saini, S. Sakai, M. A. Saleh, J. Salzwedel, S. Sambyal, V. Samsonov, L. Šándor, A. Sandoval, M. Sano, D. Sarkar, E. Scapparone, F. Scarlassara, C. Schiaua, R. Schicker, C. Schmidt, H. R. Schmidt, S. Schuchmann, J. Schukraft, M. Schulc, T. Schuster, Y. Schutz, K. Schwarz, K. Schweda, G. Scioli, E. Scomparin, R. Scott, M. Šefčík, J. E. Seger, Y. Sekiguchi, D. Sekihata, I. Selyuzhenkov, K. Senosi, S. Senyukov, E. Serradilla, A. Sevcenco, A. Shabanov, A. Shabetai, O. Shadura, R. Shahoyan, A. Shangaraev, A. Sharma, M. Sharma, M. Sharma, N. Sharma, K. Shigaki, K. Shtejer, Y. Sibiriak, S. Siddhanta, K. M. Sielewicz, T. Siemiarczuk, D. Silvermyr, C. Silvestre, G. Simatovic, G. Simonetti, R. Singaraju, R. Singh, S. Singha, V. Singhal, B. C. Sinha, T. Sinha, B. Sitar, M. Sitta, T. B. Skaali, M. Slupecki, N. Smirnov, R. J. M. Snellings, T. W. Snellman, C. Søgaard, J. Song, M. Song, Z. Song, F. Soramel, S. Sorensen, F. Sozzi, M. Spacek, E. Spiriti, I. Sputowska, M. Spyropoulou-Stassinaki, J. Stachel, I. Stan, G. Stefanek, E. Stenlund, G. Steyn, J. H. Stiller, D. Stocco, P. Strmen, A. A. P. Suaide, T. Sugitate, C. Suire, M. Suleymanov, M. Suljic, R. Sultanov, M. Šumbera, A. Szabo, A. Szanto de Toledo, I. Szarka, A. Szczepankiewicz, M. Szymanski, U. Tabassam, J. Takahashi, G. J. Tambave, N. Tanaka, M. A. Tangaro, M. Tarhini, M. Tariq, M. G. Tarzila, A. Tauro, G. Tejeda Muñoz, A. Telesca, K. Terasaki, C. Terrevoli, B. Teyssier, J. Thäder, D. Thomas, R. Tieulent, A. R. Timmins, A. Toia, S. Trogolo, G. Trombetta, V. Trubnikov, W. H. Trzaska, T. Tsuji, A. Tumkin, R. Turrisi, T. S. Tveter, K. Ullaland, A. Uras, G. L. Usai, A. Utrobicic, M. Vajzer, M. Vala, L. Valencia Palomo, S. Vallero, J. Van Der Maarel, J. W. Van Hoorne, M. van Leeuwen, T. Vanat, P. Vande Vyvre, D. Varga, A. Vargas, M. Vargyas, R. Varma, M. Vasileiou, A. Vasiliev, A. Vauthier, V. Vechernin, A. M. Veen, M. Veldhoen, A. Velure, M. Venaruzzo, E. Vercellin, S. Vergara Limón, R. Vernet, M. Verweij, L. Vickovic, G. Viesti, J. Viinikainen, Z. Vilakazi, O. Villalobos Baillie, A. Villatoro Tello, A. Vinogradov, L. Vinogradov, Y. Vinogradov, T. Virgili, V. Vislavicius, Y. P. Viyogi, A. Vodopyanov, M. A. Völkl, K. Voloshin, S. A. Voloshin, G. Volpe, B. von Haller, I. Vorobyev, D. Vranic, J. Vrláková, B. Vulpescu, A. Vyushin, B. Wagner, J. Wagner, H. Wang, M. Wang, D. Watanabe, Y. Watanabe, M. Weber, S. G. Weber, D. F. Weiser, J. P. Wessels, U. Westerhoff, A. M. Whitehead, J. Wiechula, J. Wikne, M. Wilde, G. Wilk, J. Wilkinson, M. C. S. Williams, B. Windelband, M. Winn, C. G. Yaldo, H. Yang, P. Yang, S. Yano, C. Yasar, Z. Yin, H. Yokoyama, I.-K. Yoo, J. H. Yoon, V. Yurchenko, I. Yushmanov, A. Zaborowska, V. Zaccolo, A. Zaman, C. Zampolli, H. J. C. Zanoli, S. Zaporozhets, N. Zardoshti, A. Zarochentsev, P. Závada, N. Zaviyalov, H. Zbroszczyk, I. S. Zgura, M. Zhalov, H. Zhang, X. Zhang, Y. Zhang, C. Zhang, Z. Zhang, C. Zhao, N. Zhigareva, D. Zhou, Y. Zhou, Z. Zhou, H. Zhu, J. Zhu, A. Zichichi, A. Zimmermann, M. B. Zimmermann, G. Zinovjev, M. Zyzak, ALICE Collaboration

**Affiliations:** 1 A.I. Alikhanyan National Science Laboratory (Yerevan Physics Institute) Foundation, Yerevan, Armenia; 2 Benemérita Universidad Autónoma de Puebla, Puebla, Mexico; 3 Bogolyubov Institute for Theoretical Physics, Kiev, Ukraine; 4 Department of Physics and Centre for Astroparticle Physics and Space Science (CAPSS), Bose Institute, Kolkata, India; 5 Budker Institute for Nuclear Physics, Novosibirsk, Russia; 6 California Polytechnic State University, San Luis Obispo, California USA; 7 Central China Normal University, Wuhan, China; 8 Centre de Calcul de l’IN2P3, Villeurbanne, France; 9 Centro de Aplicaciones Tecnológicas y Desarrollo Nuclear (CEADEN), Havana, Cuba; 10 Centro de Investigaciones Energéticas Medioambientales y Tecnológicas (CIEMAT), Madrid, Spain; 11 Centro de Investigación y de Estudios Avanzados (CINVESTAV), Mexico City and Mérida, Mexico; 12 Centro Fermi-Museo Storico della Fisica e Centro Studi e Ricerche “Enrico Fermi”, Rome, Italy; 13 Chicago State University, Chicago, IL USA; 14 China Institute of Atomic Energy, Beijing, China; 15 Commissariat à l’Energie Atomique, IRFU, Saclay, France; 16 COMSATS Institute of Information Technology (CIIT), Islamabad, Pakistan; 17 Departamento de Física de Partículas and IGFAE, Universidad de Santiago de Compostela, Santiago de Compostela, Spain; 18 Department of Physics and Technology, University of Bergen, Mons, Norway; 19 Department of Physics, Aligarh Muslim University, Aligarh, India; 20 Department of Physics, Ohio State University, Columbus, OH USA; 21 Department of Physics, Sejong University, Seoul, South Korea; 22 Department of Physics, University of Oslo, Oslo, Norway; 23 Dipartimento di Elettrotecnica ed Elettronica del Politecnico, Bari, Italy; 24 Dipartimento di Fisica dell’Università ‘La Sapienza’ and Sezione INFN, Rome, Italy; 25 Dipartimento di Fisica dell’Università and Sezione INFN, Cagliari, Italy; 26 Dipartimento di Fisica dell’Università and Sezione INFN, Trieste, Italy; 27 Dipartimento di Fisica dell’Università and Sezione INFN, Turin, Italy; 28 Dipartimento di Fisica e Astronomia dell’Università and Sezione INFN, Bologna, Italy; 29 Dipartimento di Fisica e Astronomia dell’Università and Sezione INFN, Catania, Italy; 30 Dipartimento di Fisica e Astronomia dell’Università and Sezione INFN, Padua, Italy; 31 Dipartimento di Fisica ‘E.R. Caianiello’ dell’Università and Gruppo Collegato INFN, Salerno, Italy; 32 Dipartimento di Scienze e Innovazione Tecnologica dell’Università del Piemonte Orientale and Gruppo Collegato INFN, Alessandria, Italy; 33 Dipartimento Interateneo di Fisica ‘M. Merlin’ and Sezione INFN, Bari, Italy; 34 Division of Experimental High Energy Physics, University of Lund, Lund, Sweden; 35 Eberhard Karls Universität Tübingen, Tübingen, Germany; 36 European Organization for Nuclear Research (CERN), Geneva, Switzerland; 37 Excellence Cluster Universe, Technische Universität München, Munich, Germany; 38 Faculty of Engineering, Bergen University College, Mons, Norway; 39 Faculty of Mathematics, Physics and Informatics, Comenius University, Bratislava, Slovakia; 40 Faculty of Nuclear Sciences and Physical Engineering, Czech Technical University in Prague, Prague, Czech Republic; 41 Faculty of Science, P.J. Šafárik University, Kosice, Slovakia; 42 Faculty of Technology, Buskerud and Vestfold University College, Vestfold, Norway; 43 Frankfurt Institute for Advanced Studies, Johann Wolfgang Goethe-Universität Frankfurt, Frankfurt, Germany; 44 Gangneung-Wonju National University, Gangneung, South Korea; 45 Department of Physics, Gauhati University, Guwahati, India; 46 Helsinki Institute of Physics (HIP), Helsinki, Finland; 47 Hiroshima University, Hiroshima, Japan; 48 Indian Institute of Technology Bombay (IIT), Mumbai, India; 49 Indian Institute of Technology Indore (IITI), Indore, India; 50 Inha University, Inchon, South Korea; 51 Institut de Physique Nucléaire d’Orsay (IPNO), Université Paris-Sud, CNRS-IN2P3, Orsay, France; 52 Institut für Informatik, Johann Wolfgang Goethe-Universität Frankfurt, Frankfurt, Germany; 53 Institut für Kernphysik, Johann Wolfgang Goethe-Universität Frankfurt, Frankfurt, Germany; 54 Institut für Kernphysik, Westfälische Wilhelms-Universität Münster, Münster, Germany; 55 Institut Pluridisciplinaire Hubert Curien (IPHC), Université de Strasbourg, CNRS-IN2P3, Strasbourg, France; 56 Institute for Nuclear Research, Academy of Sciences, Moscow, Russia; 57 Institute for Subatomic Physics of Utrecht University, Utrecht, The Netherlands; 58 Institute for Theoretical and Experimental Physics, Moscow, Russia; 59 Institute of Experimental Physics, Slovak Academy of Sciences, Kosice, Slovakia; 60 Institute of Physics, Academy of Sciences of the Czech Republic, Prague, Czech Republic; 61 Institute of Physics, Bhubaneswar, India; 62 Institute of Space Science (ISS), Bucharest, Romania; 63 Instituto de Ciencias Nucleares, Universidad Nacional Autónoma de México, Mexico City, Mexico; 64 Instituto de Física, Universidad Nacional Autónoma de México, Mexico City, Mexico; 65 iThemba LABS, National Research Foundation, Somerset West, South Africa; 66 Joint Institute for Nuclear Research (JINR), Dubna, Russia; 67 Konkuk University, Seoul, South Korea; 68 Korea Institute of Science and Technology Information, Taejon, South Korea; 69 KTO Karatay University, Konya, Turkey; 70 Laboratoire de Physique Corpusculaire (LPC), Clermont Université, Université Blaise Pascal, CNRS-IN2P3, Clermont-Ferrand, France; 71 Laboratoire de Physique Subatomique et de Cosmologie, Université Grenoble-Alpes, CNRS-IN2P3, Grenoble, France; 72 Laboratori Nazionali di Frascati, INFN, Frascati, Italy; 73 Laboratori Nazionali di Legnaro, INFN, Legnaro, Italy; 74 Lawrence Berkeley National Laboratory, Berkeley, CA USA; 75 Moscow Engineering Physics Institute, Moscow, Russia; 76 Nagasaki Institute of Applied Science, Nagasaki, Japan; 77 National Centre for Nuclear Studies, Warsaw, Poland; 78 National Institute for Physics and Nuclear Engineering, Bucharest, Romania; 79 National Institute of Science Education and Research, Bhubaneswar, India; 80 National Research Centre Kurchatov Institute, Moscow, Russia; 81 Niels Bohr Institute, University of Copenhagen, Copenhagen, Denmark; 82 Nikhef, Nationaal instituut voor subatomaire fysica, Amsterdam, The Netherlands; 83 Nuclear Physics Group, STFC Daresbury Laboratory, Daresbury, UK; 84 Nuclear Physics Institute, Academy of Sciences of the Czech Republic, Řež u Prahy, Czech Republic; 85 Oak Ridge National Laboratory, Oak Ridge, TN USA; 86 Petersburg Nuclear Physics Institute, Gatchina, Russia; 87 Physics Department, Creighton University, Omaha, NE USA; 88 Physics Department, Panjab University, Chandigarh, India; 89 Physics Department, University of Athens, Athens, Greece; 90 Physics Department, University of Cape Town, Cape Town, South Africa; 91 Physics Department, University of Jammu, Jammu, India; 92 Physics Department, University of Rajasthan, Jaipur, India; 93 Physik Department, Technische Universität München, Munich, Germany; 94 Physikalisches Institut, Ruprecht-Karls-Universität Heidelberg, Heidelberg, Germany; 95 Purdue University, West Lafayette, IN USA; 96 Pusan National University, Pusan, South Korea; 97 Research Division and ExtreMe Matter Institute EMMI, GSI Helmholtzzentrum für Schwerionenforschung, Darmstadt, Germany; 98 Rudjer Bošković Institute, Zagreb, Croatia; 99 Russian Federal Nuclear Center (VNIIEF), Sarov, Russia; 100 Saha Institute of Nuclear Physics, Kolkata, India; 101 School of Physics and Astronomy, University of Birmingham, Birmingham, UK; 102 Sección Física, Departamento de Ciencias, Pontificia Universidad Católica del Perú, Lima, Peru; 103 Sezione INFN, Bari, Italy; 104 Sezione INFN, Bologna, Italy; 105 Sezione INFN, Cagliari, Italy; 106 Sezione INFN, Catania, Italy; 107 Sezione INFN, Padua, Italy; 108 Sezione INFN, Rome, Italy; 109 Sezione INFN, Trieste, Italy; 110 Sezione INFN, Turin, Italy; 111 SSC IHEP of NRC Kurchatov institute, Protvino, Russia; 112 Stefan Meyer Institut für Subatomare Physik (SMI), Vienna, Austria; 113 SUBATECH, Ecole des Mines de Nantes, Université de Nantes, CNRS-IN2P3, Nantes, France; 114 Suranaree University of Technology, Nakhon Ratchasima, Thailand; 115 Technical University of Košice, Kosice, Slovakia; 116 Technical University of Split FESB, Split, Croatia; 117 The Henryk Niewodniczanski Institute of Nuclear Physics, Polish Academy of Sciences, Kraków, Poland; 118 Physics Department, The University of Texas at Austin, Austin, TX USA; 119 Universidad Autónoma de Sinaloa, Culiacán, Mexico; 120 Universidade de São Paulo (USP), São Paulo, Brazil; 121 Universidade Estadual de Campinas (UNICAMP), Campinas, Brazil; 122 University of Houston, Houston, TX USA; 123 University of Jyväskylä, Jyväskylä, Finland; 124 University of Liverpool, Liverpool, UK; 125 University of Tennessee, Knoxville, TN USA; 126 University of the Witwatersrand, Johannesburg, South Africa; 127 University of Tokyo, Tokyo, Japan; 128 University of Tsukuba, Tsukuba, Japan; 129 University of Zagreb, Zagreb, Croatia; 130 Université de Lyon, Université Lyon 1, CNRS/IN2P3, IPN-Lyon, Villeurbanne, France; 131 V. Fock Institute for Physics, St. Petersburg State University, St. Petersburg, Russia; 132 Variable Energy Cyclotron Centre, Kolkata, India; 133 Warsaw University of Technology, Warsaw, Poland; 134 Wayne State University, Detroit, MI USA; 135 Wigner Research Centre for Physics, Hungarian Academy of Sciences, Budapest, Hungary; 136 Yale University, New Haven, CT USA; 137 Yonsei University, Seoul, South Korea; 138 Zentrum für Technologietransfer und Telekommunikation (ZTT), Fachhochschule Worms, Worms, Germany; 139 CERN, Geneva, Switzerland

## Abstract

We report on the inclusive production cross sections of ${\mathrm{J}/\psi }$, ${\psi (\mathrm{2S})}$, $\mathrm{\Upsilon }$(1S), $\mathrm{\Upsilon }$(2S) and $\mathrm{\Upsilon }$(3S), measured at forward rapidity with the ALICE detector in $\mathrm{pp}$ collisions at a center-of-mass energy $\sqrt{s}=8$ TeV. The analysis is based on data collected at the LHC and corresponds to an integrated luminosity of 1.23 pb$^{-1}$. Quarkonia are reconstructed in the dimuon-decay channel. The differential production cross sections are measured as a function of the transverse momentum ${p_\mathrm{T}}$ and rapidity *y*, over the ${p_\mathrm{T}}$ ranges $0<{p_\mathrm{T}}<20$ GeV/*c* for ${\mathrm{J}/\psi }$, $0<{p_\mathrm{T}}<12$ GeV/*c* for all other resonances, and for $2.5<y<4$. The cross sections, integrated over ${p_\mathrm{T}}$ and *y*, and assuming unpolarized quarkonia, are $\sigma _{{\mathrm{J}/\psi }} = 8.98\pm 0.04\pm 0.82$ $\upmu$b, $\sigma _{{\psi (\mathrm{2S})}} = 1.23\pm 0.08\pm 0.22$ $\upmu$b, $\sigma _{\mathrm{\Upsilon }\mathrm{(1S)}} = 71\pm 6\pm 7$ nb, $\sigma _{\mathrm{\Upsilon }\mathrm{(2S)}} = 26\pm 5\pm 4$ nb and $\sigma _{\mathrm{\Upsilon }\mathrm{(3S)}} = 9\pm 4\pm 1$ nb, where the first uncertainty is statistical and the second one is systematic. These values agree, within at most $1.4\sigma$, with measurements performed by the LHCb collaboration in the same rapidity range.

## Introduction

The hadronic production of quarkonia, bound states of either a charm and anti-charm quark pair (e.g. ${\mathrm{J}/\psi }$ and ${\psi (\mathrm{2S})}$) or a bottom and anti-bottom quark pair (e.g. $\mathrm{\Upsilon }$(1S), $\mathrm{\Upsilon }$(2S) and $\mathrm{\Upsilon }$(3S)), is generally understood as the result of a hard scattering that produces the heavy-quark pair, followed by the evolution of this pair into a colorless bound state. There are mainly three approaches used to describe quarkonium production, which differ mostly in the way the produced heavy-quark pair evolves into the bound state: the Color Evaporation Model [1, 2], the Color Singlet Model [3] and Non-Relativistic QCD [4]. To date, none of these approaches is able to describe consistently all data available on quarkonium production [5, 6].

In this paper we present the production cross sections of ${\mathrm{J}/\psi }$, ${\psi (\mathrm{2S})}$, $\mathrm{\Upsilon }$(1S), $\mathrm{\Upsilon }$(2S) and $\mathrm{\Upsilon }$(3S) at forward rapidity ($2.5<y<4$), measured in pp collisions at a center-of-mass energy $\sqrt{s}=8$ TeV with the ALICE detector. All quarkonia are reconstructed in the dimuon-decay channel. The differential production cross sections are measured as a function of the transverse momentum ${p_\mathrm{T}}$ and rapidity *y*, over the ${p_\mathrm{T}}$ ranges $0<{p_\mathrm{T}}<20$ GeV/*c* for ${\mathrm{J}/\psi }$, $0<{p_\mathrm{T}}<12$ GeV/*c* for all other resonances, and for $2.5<y<4$. Our measurement extends the transverse momentum reach of the ${\mathrm{J}/\psi }$ cross section from ${p_\mathrm{T}}=12$ GeV/*c* up to ${p_\mathrm{T}}= 20$ GeV/*c* with respect to results from LHCb [7]. The ${\psi (\mathrm{2S})}$ results are the first published at this energy. For $\mathrm{\Upsilon }$ mesons, differential cross sections at forward rapidity and $\sqrt{s}=8$ TeV have already been published by LHCb [8]. Our measurement provides a unique cross-check of these results. Moreover, it is the first time ALICE measures the $\mathrm{\Upsilon }$(3S) cross section. All cross sections reported here are inclusive and contain, on top of the direct production of the quarkonium, a contribution from the decay of higher-mass excited states. Charmonium (${\mathrm{J}/\psi }$ and ${\psi (\mathrm{2S})}$) cross sections also contain a contribution from *b*-hadron decay.

The paper is organized as follows: the ALICE detector and the data sample used for this analysis are briefly described in Sect. 2, the analysis procedure is discussed in Sect. 3 and results are presented in Sect. 4.

## Detector and data sample

The ALICE detector is described in [9] and its performance in [10]. The following subsystems are used for measuring the quarkonium production cross sections at forward rapidity: the Muon Spectrometer [11], the first two layers of the Inner Tracking System (ITS) [12], the V0 scintillator hodoscopes [13] and the T0 Cherenkov counters [14].

The Muon Spectrometer consists of five tracking stations (MCH) comprising two planes of Cathode Pad Chambers each, followed by two trigger stations (MTR) consisting of two planes of Resistive Plate Chambers each. It is used to detect muons produced in the pseudo-rapidity range $-4<\eta <-2.5$ 1. The third tracking station is located inside a warm 3 T m dipole magnet, to allow for momentum measurements. This apparatus is completed by two absorbers that filter out hadrons and low ${p_\mathrm{T}}$ muons, positioned (i) between the Interaction Point (IP) and the first tracking station, and (ii) between the last tracking station and the first trigger station. A third absorber, surrounding the beam pipe, protects the detectors from secondary particles produced inside the beam pipe. The MTR system delivers single- or di-muon triggers, of either same or opposite sign, with a programmable threshold on the transverse momentum of each muon. The ITS consists of 6 layers of silicon detectors, placed at radii ranging from 3.9 to 43 cm from the beam axis. Its two innermost layers are equipped with Silicon Pixel Detectors (SPD) and cover the pseudo-rapidity ranges $|\eta |<2$ and $|\eta |<1.4$ for the inner and the outer layer, respectively. They are used for the reconstruction of the collision primary vertex. The V0 detectors are two scintillator arrays located on both sides of the IP and covering the pseudo-rapidity ranges $-3.7<\eta <-1.7$ and $2.8<\eta <5.1$. The T0 detectors are two arrays of quartz Cherenkov counters, also placed at forward rapidity on both sides of the IP and covering the pseudo-rapidity ranges $-3.3<\eta <-3$ and $4.6<\eta <4.9$. The coincidence of a signal in both sides of either the T0 or the V0 detectors is used as an interaction trigger and as input for the luminosity determination.

The data used for this analysis have been collected in 2012. About 1400 proton bunches were circulating in each LHC beam. Collisions were delivered in a so-called beam-satellite mode, for which the high-intensity bunches of one of the two beams were collided with nearly-empty satellite bunches from the other [10]. In this configuration, the average instantaneous luminosity delivered by the LHC to ALICE was about $5\times 10^{30}$ cm$^{-2}$ s$^{-1}$. The number of interactions per bunch-satellite crossing was about 0.01 on average with a corresponding pile-up probability of about 0.5 %, reaching a maximum of $\sim$1 %.

Events are selected using a dimuon trigger which requires that two muons of opposite sign are detected in the MTR, with a threshold of 1 GeV/*c* applied online to the ${p_\mathrm{T}}$ of each muon, in coincidence with the crossing of two bunches at the IP. The data sample recorded with this trigger corresponds to an integrated luminosity ${L_\mathrm{int}}= 1.23$ pb$^{-1}$. It is evaluated on a run-by-run basis by multiplying the dimuon trigger live-time with the delivered luminosity. The latter is estimated using the number of T0-based trigger counts and the corresponding cross section, $\sigma _\mathrm{T0}$, measured using the van der Meer scan method [15]. The systematic uncertainty on this quantity includes contributions from (i) the measurement of $\sigma _\mathrm{T0}$ itself and (ii) the difference between the luminosity measured with the T0 detectors and the one measured with the V0 detectors. The quadratic sum of these contributions amounts to about 5 % and is correlated between all measurements presented in this paper.

## Analysis

The differential quarkonium production cross section in a given ${p_\mathrm{T}}$ and *y* interval is:

1 $$\begin{aligned} \frac{d^2\sigma }{d{p_\mathrm{T}}dy}=\frac{1}{\Delta {p_\mathrm{T}}\Delta y}\frac{1}{{L_\mathrm{int}}}\frac{N}{\mathrm{BR}_{\mu \mu }{A\epsilon }}, \end{aligned}$$

where $\mathrm{BR}_{\mu \mu }$ is the branching ratio of the quarkonium state in two muons, $\Delta {p_\mathrm{T}}$ and $\Delta y$ are the widths of the ${p_\mathrm{T}}$ and *y* intervals under consideration, *N* is the measured number of quarkonia in these intervals and ${A\epsilon }$ is the product of the corresponding acceptance and efficiency corrections, which account for detector effects and analysis cuts. The branching ratio values and uncertainties have been taken from the Particle Data Group (PDG) [16]. The other ingredients, namely *N* and ${A\epsilon }$, have been evaluated using the analysis procedure described in [17].

The number of quarkonia measured in a given ${p_\mathrm{T}}$ and *y* interval is evaluated using fits to the invariant mass distribution of opposite-sign muon pairs ${\mu ^+\mu ^-}$. These pairs are formed by combining the tracks reconstructed in the muon spectrometer and selected using the same criteria as in [17]:

- muon identification is performed by matching each track reconstructed in the MCH with a track in the MTR that fulfills the trigger condition;
- tracks are selected in the pseudo-rapidity range $-4<\eta <-2.5$, which corresponds to the muon spectrometer geometrical acceptance;
- the transverse position of the tracks at the end of the front absorber, $R_\mathrm{{abs}}$, is in the range $17.6 < R_\mathrm{{abs}} < 89.5$ cm, in order to reject muons crossing the high-density section of the front absorber;
- tracks must pass a cut on the product of their total momentum, *p*, and their distance to the primary vertex in the transverse plane, called DCA. The maximum value allowed is set to $6\times \sigma _{p}\mathrm{DCA}$, where $\sigma _{p}\mathrm{DCA}$ is the resolution on this quantity, which accounts for the total momentum and angular resolutions of the muon spectrometer as well as for the multiple scattering in the front absorber. This cut reduces the contamination of fake tracks and particles from beam-gas interactions.

The fit to the ${\mu ^+\mu ^-}$ invariant mass distribution is performed separately in the charmonium and bottomonium regions, and for each ${p_\mathrm{T}}$ and *y* interval under consideration. In all cases the fitting function consists of a background to which two (three) signal functions are added, one per charmonium (bottomonium) state under study.

**Fig. 1:**
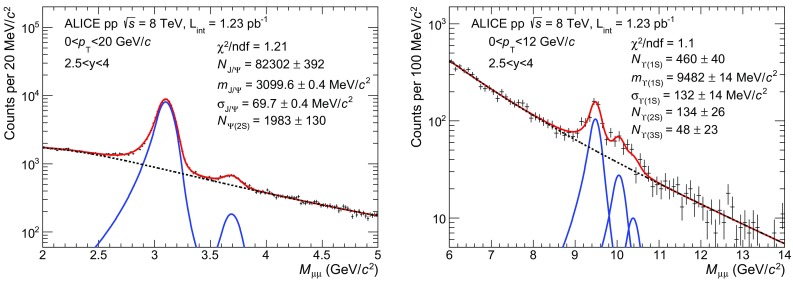
Dimuon invariant mass distributions in the region of charmonia (*left*) and bottomonia (*right*). *Dashed lines* correspond to the background. *Solid lines* correspond to either the signal functions, or the sum of all signal and background functions. In the charmonia region, the sum of two extended Crystal Ball functions is used for the signal and a pseudo-Gaussian function is used for the background. In the bottomonia region, the sum of three extended Crystal Ball functions is used for the signal and the sum of two exponential functions is used for the background

For charmonia, the fit is performed over the invariant mass range $2<{M_{\mu \mu }}<5$ GeV/$c^2$. For the background component, either a pseudo-Gaussian function whose width varies linearly with the invariant mass or the product of an exponential function and a fourth order polynomial function have been used, with all parameters left free in the fit. For the signal, the sum of either two extended Crystal Ball functions (one for each resonance) or two pseudo-Gaussian functions have been used [18]. Both functions (Crystal Ball or pseudo-Gaussian) consist of a Gaussian core, to which parametrized tails are added on both sides, which fall off slower than for a Gaussian function. Due to the poor signal-to-background (S/B) ratio in the tail regions, the values of the parameters that enter the definition of these tails have been evaluated using Monte Carlo (MC) simulations described later in this section, and kept fixed in the fit. The ${\mathrm{J}/\psi }$ and ${\psi (\mathrm{2S})}$ signals are fitted simultaneously. For the ${\mathrm{J}/\psi }$, the mass, width and normalization of the signal function are left free. For the ${\psi (\mathrm{2S})}$, only the normalization is free, whereas the mass and the width are calculated from the values obtained for the ${\mathrm{J}/\psi }$: the mass is computed so that the difference with respect to the ${\mathrm{J}/\psi }$ mass is the same as quoted by the PDG [16]; the width is derived from the ${\mathrm{J}/\psi }$ width using a scale factor of about 1.1, estimated in MC simulations and validated with fits to the ${p_\mathrm{T}}$- and *y*-integrated invariant mass distributions from the data, with both widths left free. An example of fit to the ${p_\mathrm{T}}$- and *y*-integrated dimuon invariant mass distribution in the ${\mathrm{J}/\psi }$ and ${\psi (\mathrm{2S})}$ mass region is shown in the left panel of Fig. 1. The result from this fit is used for the computation of the charmonium cross sections quoted at the beginning of Sect. 4.

For the $\mathrm{\Upsilon }$ resonances, the fit is performed over the invariant mass range $6<{M_{\mu \mu }}<14$ GeV/$c^2$. The same signal functions as for the ${\mathrm{J}/\psi }$ and ${\psi (\mathrm{2S})}$ have been used for each of the three resonances, albeit with different values for the parameters of the tails. For the background component, either the sum of two exponential functions or the sum of two power law functions have been used, with all parameters left free. The masses and widths of the $\mathrm{\Upsilon }$(2S) and $\mathrm{\Upsilon }$(3S) resonances have been fixed to the ones of the $\mathrm{\Upsilon }$(1S) in a similar way as for the ${\psi (\mathrm{2S})}$ and ${\mathrm{J}/\psi }$ case, and using a similar scale factor for the width. An example of fit to the ${p_\mathrm{T}}$- and *y*-integrated dimuon invariant mass distribution in the $\mathrm{\Upsilon }$ mass region is shown in the right panel of Fig. 1.

The number of quarkonia is taken as the mean of the values obtained when (i) combining all possible signal and background functions described above; (ii) varying the parameters that have been fixed, such as those of the tails of the signal functions or the ratio between the ${\psi (\mathrm{2S})}$ and the ${\mathrm{J}/\psi }$ signal widths, and (iii) modifying the mass range used for the fit.

Approximately 82500 ${\mathrm{J}/\psi }$, 1850 ${\psi (\mathrm{2S})}$, 480 $\mathrm{\Upsilon }$(1S), 140 $\mathrm{\Upsilon }$(2S) and 50 $\mathrm{\Upsilon }$(3S) are measured. The corresponding S/B ratios, evaluated within three times the width of the signal function with respect to the quarkonium mass are 4.5 for ${\mathrm{J}/\psi }$, 0.2 for ${\psi (\mathrm{2S})}$, 1 for $\mathrm{\Upsilon }$(1S), 0.4 for $\mathrm{\Upsilon }$(2S) and 0.2 for $\mathrm{\Upsilon }$(3S). This statistics allows us to divide the data sample further as a function of either ${p_\mathrm{T}}$ or *y* for ${\mathrm{J}/\psi }$, ${\psi (\mathrm{2S})}$ and $\mathrm{\Upsilon }$(1S). For $\mathrm{\Upsilon }$(2S), only two bins in *y* are measured, whereas for $\mathrm{\Upsilon }$(3S), only the ${p_\mathrm{T}}$- and *y*-integrated value is provided, due to limited statistics. For ${\mathrm{J}/\psi }$, the S/B ratio increases from 3 to 10 with increasing ${p_\mathrm{T}}$ and from 4 to 6 with increasing *y*. For ${\psi (\mathrm{2S})}$, it increases from 0.1 to 0.9 with increasing ${p_\mathrm{T}}$ and from 0.1 to 0.2 with increasing *y*. For $\mathrm{\Upsilon }$(1S), it increases from 0.8 to 1.4 with increasing ${p_\mathrm{T}}$ and shows no significant variation with respect to *y*. No significant variation with respect to *y* is observed for $\mathrm{\Upsilon }$(2S) either.

The systematic uncertainty on the signal extraction is estimated by taking the root mean square of the values from which the number of quarkonia is derived. For a given quarkonium state, this uncertainty is considered as uncorrelated as a function of both ${p_\mathrm{T}}$ and *y*. It is however partially correlated between ${\mathrm{J}/\psi }$ and ${\psi (\mathrm{2S})}$ as well as among the three resonances of the $\mathrm{\Upsilon }$ family. For ${\mathrm{J}/\psi }$ this uncertainty increases from less than 1 to 14 % with increasing ${p_\mathrm{T}}$. It shows no significant variation with respect to *y* and amounts to about 1 %. Larger values are obtained for ${\psi (\mathrm{2S})}$ due to the smaller S/B ratio. For instance, the uncertainty reaches 18 % in the *y* interval $2.5<y<2.75$. In the $\mathrm{\Upsilon }$ sector, the systematic uncertainty is about 3, 6 and 10 % for $\mathrm{\Upsilon }$(1S), $\mathrm{\Upsilon }$(2S) and $\mathrm{\Upsilon }$(3S), respectively, with little variation as a function of either ${p_\mathrm{T}}$ or *y*.

Acceptance and efficiency corrections, ${A\epsilon }$, are evaluated separately for each quarkonium state using MC simulations. Each state is generated randomly using realistic ${p_\mathrm{T}}$ and *y* probability distribution functions [11, 17]. It is decayed in two muons, properly accounting for the possible emission of an accompanying radiative photon [19, 20]. The muons are then tracked in a model of the apparatus obtained with GEANT 3.21 [21] which includes a realistic description of the detector performance during data taking as well as its variation with time. The same procedure and analysis cuts as for data are then applied to the MC simulations for track reconstruction and measurement of the quarkonium yields. All simulated quarkonia are assumed to be unpolarized, consistently with existing measurements [22–25].

The systematic uncertainty on ${A\epsilon }$ has several contributions: (i) the parametrization of the input ${p_\mathrm{T}}$ and *y* distributions; (ii) the track reconstruction efficiency and the accuracy with which the detector performance is reproduced in the MC simulations; (iii) the trigger efficiency and (iv) the matching between tracks reconstructed in the MCH and tracks reconstructed in the MTR. These contributions have been evaluated using the same procedures as in [17], for the first one by utilizing several alternative input ${p_\mathrm{T}}$ and *y* distributions, and for the other three by comparing data and MC at the single muon level and propagating the resulting differences to the dimuon case. The resulting systematic uncertainty is the quadratic sum of these contributions. It is partially correlated as a function of both ${p_\mathrm{T}}$ and *y*. For all quarkonium states, it amounts to about 8 % on average, increases from 7 to 9 % with increasing ${p_\mathrm{T}}$ and shows no visible dependence on *y*.

An additional correction is applied to the number of measured quarkonia, to account for the observation that a fraction of the opposite-sign muon pairs of a given quarkonium state is sometimes misidentified by the trigger system as a same-sign pair and thus missed. The magnitude of this effect could not be properly reproduced in the MC simulations and is therefore not accounted for in the ${A\epsilon }$ corrections. For ${\mathrm{J}/\psi }$ and $\mathrm{\Upsilon }$(1S), it is instead evaluated directly on data by means of a dedicated trigger configuration that selects both same- and opposite-sign muon pairs instead of opposite-sign pairs only. The statistical and systematic uncertainties on the extraction of the signal in each configuration are used to evaluate the systematic uncertainty on the resulting correction. For ${\mathrm{J}/\psi }$, the correction amounts to about 1 % on the ${p_\mathrm{T}}$- and *y*-integrated yield. It increases from 0.6 % to 8 % with increasing ${p_\mathrm{T}}$ and shows little dependence on *y*. Slightly larger values are obtained for $\mathrm{\Upsilon }$(1S) albeit with larger uncertainties. For ${\psi (\mathrm{2S})}$, the same corrections as for ${\mathrm{J}/\psi }$ have been used, whereas for $\mathrm{\Upsilon }$(2S) and $\mathrm{\Upsilon }$(3S) we used the same corrections as for $\mathrm{\Upsilon }$(1S).

Tables 1 and 2 provide a summary of the relative systematic uncertainties on the charmonia and bottomonia cross sections, respectively.

**Table 1:** Relative systematic uncertainties associated to the ${\mathrm{J}/\psi }$ and ${\psi (\mathrm{2S})}$ cross section measurements. Values in parenthesis correspond to minimum and maximum values as a function of ${p_\mathrm{T}}$ and *y*

Source	${\mathrm{J}/\psi }$ (%)	${\psi (\mathrm{2S})}$ (%)
Luminosity	5	5
Branching ratio	$<$1	11
Signal extraction	1 ($<$1–14)	10 (6–18)
Acceptance$\times$efficiency	8 (7–9)	8 (7–9)
Trigger sign	$<1$ ($<$1–3)	$<$1 ($<$1–3)

**Table 2:** Relative systematic uncertainties associated to the $\mathrm{\Upsilon }$(1S), $\mathrm{\Upsilon }$(2S) and $\mathrm{\Upsilon }$(3S) cross section measurements. Values in parenthesis correspond to minimum and maximum values as a function of ${p_\mathrm{T}}$ and *y*

Source	$\mathrm{\Upsilon }$(1S) (%)	$\mathrm{\Upsilon }$(2S) (%)	$\mathrm{\Upsilon }$(3S) (%)
Luminosity	5	5	5
Branching ratio	2	9	10
Signal extraction	3 (2–6)	6 (5–9)	10
Acceptance$\times$efficiency	8 (7–9)	8	8
Trigger sign	1 (1–5)	1 (1–2)	1

## Results

The measured inclusive quarkonium production cross sections, integrated over $0<{p_\mathrm{T}}<20$ GeV/*c* for ${\mathrm{J}/\psi }$, $0<{p_\mathrm{T}}<12$ GeV/*c* for all other resonances, and $2.5<y<4$, are:

$$\sigma _{{\mathrm{J}/\psi }} = 8.98\pm 0.04\mathrm{(stat)}\pm 0.82\mathrm{(syst)}$$ $$\upmu $$b,

$$\sigma _{{\psi (\mathrm{2S})}} = 1.23\pm 0.08\mathrm{(stat)}\pm 0.22\mathrm{(syst)}$$ $$\upmu $$b,

$$\sigma _{\mathrm{\Upsilon }\mathrm{(1S)}} = 71\pm 6\mathrm{(stat)}\pm 7\mathrm{(syst)}$$ nb,

$$\sigma _{\mathrm{\Upsilon }\mathrm{(2S)}} = 26\pm 5\mathrm{(stat)}\pm 4\mathrm{(syst)}$$ nb and

$$\sigma _{\mathrm{\Upsilon }\mathrm{(3S)}} = 9\pm 4\mathrm{(stat)}\pm 1\mathrm{(syst)}$$ nb.

These values are in agreement, within at most $1.4\sigma$, with measurements performed by LHCb at the same energy and in the same rapidity range [7, 8], assuming that all uncertainties but the one on the branching ratios are uncorrelated between the two experiments. For ${\mathrm{J}/\psi }$, our cross section value corresponds to an increase of $(29\pm 17)$ % with respect to the ALICE measurement at $\sqrt{s}=7$ TeV [17]. A similar increase is observed for ${\psi (\mathrm{2S})}$ and for the $\mathrm{\Upsilon }$ resonances, albeit with larger uncertainties.

**Fig. 2:**
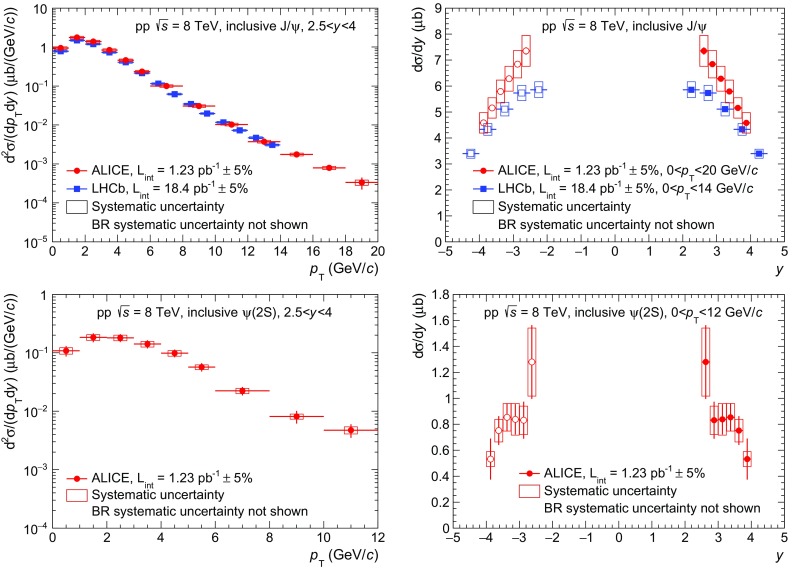
${\mathrm{J}/\psi }$ (*top*) and ${\psi (\mathrm{2S})}$ (*bottom*) differential cross sections as a function of ${p_\mathrm{T}}$ (*left*) and *y* (*right*). ${\mathrm{J}/\psi }$ results are compared to LHCb measurement at $\sqrt{s}=8$ TeV [7]. *Open symbols* are the reflection of the positive-*y* measurements with respect to $y=0$. *Vertical error bars* are the statistical uncertainties. *Boxes* are the systematic uncertainties. Branching ratio uncertainties are not included

Figure 2 shows the inclusive differential production cross sections of ${\mathrm{J}/\psi }$ (top) and ${\psi (\mathrm{2S})}$ (bottom) as a function of ${p_\mathrm{T}}$ (left) and *y* (right) in pp collisions at $\sqrt{s}=8$ TeV. In all the plots, the error bars represent the statistical uncertainties and the boxes correspond to the systematic uncertainties. Branching ratio uncertainties are not included. The ${\mathrm{J}/\psi }$ ${p_\mathrm{T}}$- and *y*-differential cross sections are compared to measurements by LHCb at the same energy [7]. The quoted LHCb values correspond to the sum of the prompt and *b*-meson decay contributions to the ${\mathrm{J}/\psi }$ production. For the comparison as a function of ${p_\mathrm{T}}$, the provided double-differential (${p_\mathrm{T}}$ and *y*) values have been re-summed to match ALICE *y* coverage. A reasonable agreement is observed between the two experiments. Although the ALICE measurements are systematically above those of LHCb especially at low ${p_\mathrm{T}}$ and small |*y*|, in both cases the differences do not exceed $1.7\sigma$. The ALICE measurement extends the ${p_\mathrm{T}}$ reach of the ${\mathrm{J}/\psi }$ cross section from 14 GeV/*c* to 20 GeV/*c* with respect to published results. The ${\psi (\mathrm{2S})}$ cross sections constitute the first measurement performed at this energy.

**Fig. 3:**
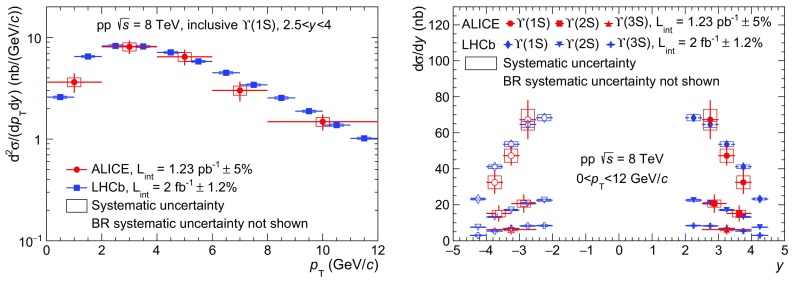
Differential cross section of $\mathrm{\Upsilon }$(1S) as a function of ${p_\mathrm{T}}$ (*left*) and differential cross sections of $\mathrm{\Upsilon }$(1S), $\mathrm{\Upsilon }$(2S) and $\mathrm{\Upsilon }$(3S) as a function of *y* (*right*) measured by ALICE and LHCb [8]. *Open symbols* are the reflection of the positive-*y* measurements with respect to $y=0$

Figure 3 shows the inclusive differential production cross sections of $\mathrm{\Upsilon }$(1S) as a function of ${p_\mathrm{T}}$ (left) and of the $\mathrm{\Upsilon }$(1S), $\mathrm{\Upsilon }$(2S) and $\mathrm{\Upsilon }$(3S) as a function of *y* (right). Results are compared to measurements by LHCb at the same energy [8]. For the comparison as a function of ${p_\mathrm{T}}$ (resp. *y*), the double-differential values provided by LHCb have been re-summed to match the *y* (resp. ${p_\mathrm{T}}$) range of ALICE. Moreover, although the ${p_\mathrm{T}}$ range measured by LHCb extends to values as large as 30 GeV/*c*, we only show these measurements in the range $0<{p_\mathrm{T}}<12$ GeV/*c*, which is more relevant for the comparison to our result. A reasonable agreement is observed between the two experiments. For $\mathrm{\Upsilon }$(1S), ALICE measurements are systematically lower than those from LHCb, however the differences do not exceed $1.2\sigma$ as a function of either ${p_\mathrm{T}}$ or *y*.

The inclusive ${\psi (\mathrm{2S})}$-to-${\mathrm{J}/\psi }$ cross section ratio at $\sqrt{s}=8$ TeV, integrated over ${p_\mathrm{T}}$ and *y* is $\sigma _{\psi (\mathrm{2S})}/ \sigma _{\mathrm{J}/\psi }= 0.14\pm 0.01\pm 0.02$, the $\mathrm{\Upsilon }$(2S)-to-$\mathrm{\Upsilon }$(1S) ratio is $\sigma _{\mathrm{\Upsilon }\mathrm (2S)} / \sigma _{\mathrm{\Upsilon }\mathrm (1S)} = 0.37\pm 0.08\pm 0.04$ and the $\mathrm{\Upsilon }$(3S)-to-$\mathrm{\Upsilon }$(1S) ratio, $\sigma _{\mathrm{\Upsilon }\mathrm (3S)} / \sigma _{\mathrm{\Upsilon }\mathrm (1S)} = 0.12\pm 0.05\pm 0.02$, where the first uncertainty is statistical and the second one is systematic. When forming these ratios, the systematic uncertainty on the signal extraction is slightly reduced, due to correlations between the numerator and the denominator. All other sources of systematic uncertainties cancel, except for the uncertainties on the input ${p_\mathrm{T}}$ and *y* parametrizations in the MC, and on $\mathrm{BR}_{\mu \mu }$. The ${\psi (\mathrm{2S})}$-to-${\mathrm{J}/\psi }$ and $\mathrm{\Upsilon }$(2S)-to-$\mathrm{\Upsilon }$(1S) ratios are consistent with the values obtained in the same rapidity range at $\sqrt{s}=7$ TeV [17].

**Fig. 4:**
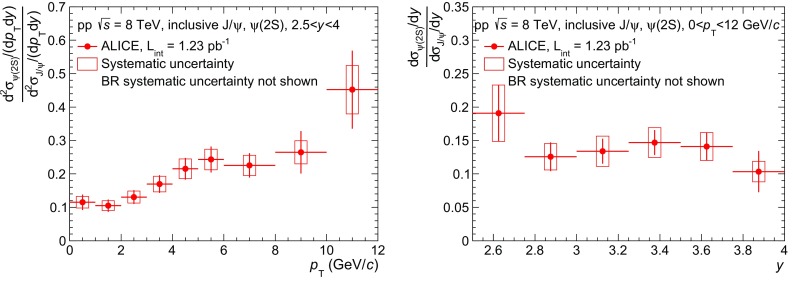
${\psi (\mathrm{2S})}$-to-${\mathrm{J}/\psi }$ cross section ratio as a function of ${p_\mathrm{T}}$ (*left*) and *y* (*right*)

Figure 4 shows the ${\psi (\mathrm{2S})}$-to-${\mathrm{J}/\psi }$ cross section ratio as a function of ${p_\mathrm{T}}$ (left) and *y* (right). This ratio increases as a function of ${p_\mathrm{T}}$ with a slope that is similar to the one measured at $\sqrt{s}=7$ TeV [17]. It shows no visible variation as a function of *y*, as was also the case at 7 TeV.

## Conclusion

The inclusive production cross section of ${\mathrm{J}/\psi }$, ${\psi (\mathrm{2S})}$, $\mathrm{\Upsilon }$(1S), $\mathrm{\Upsilon }$(2S) and $\mathrm{\Upsilon }$(3S) as a function of ${p_\mathrm{T}}$ and *y* have been measured using the ALICE detector at forward rapidity ($2.5<y<4$) in pp collisions at $\sqrt{s}=8$ TeV. The ${\mathrm{J}/\psi }$ cross section is larger by $(29\pm 17)$ % than the one measured at $\sqrt{s}=7$ TeV [17]. A similar increase is observed for the other quarkonium states albeit with larger uncertainties. The integrated results are in agreement within at most $1.4\sigma$ with measurements performed by LHCb in the same rapidity range. For the differential measurements, differences with LHCb do not exceed $1.7\sigma$ for charmonia and $1.2\sigma$ for bottomonia. These measurements provide a valuable cross-check of the already published results of the same quantities as well as additional experimental constraints on quarkonium production models.

## Footnotes

1 In the ALICE reference frame the muon spectrometer covers negative $\eta$. However, we use positive values when referring to the quarkonium rapidity *y*.
